# Monte Carlo simulation of the system performance of a long axial field-of-view PET based on monolithic LYSO detectors

**DOI:** 10.1186/s40658-023-00559-2

**Published:** 2023-06-13

**Authors:** Maya Abi-Akl, Meysam Dadgar, Yassine Toufique, Othmane Bouhali, Stefaan Vandenberghe

**Affiliations:** 1 Department of Electronics and Information Systems, Medical Image and Signal Processing, Ghent University, Ghent, Belgium; 2 Division of Arts and Sciences, Texas A&M University at Qatar, Doha, Qatar; 3 Energy, Materials, Numerical Physics, Ecole Normal Supérieur (ENS), Abdelmalek Essaadi University, Tétouan, Morocco

**Keywords:** Total-body positron emission tomography, Monolithic detector, Spatial resolution, NEMA performance

## Abstract

**Background:**

In light of the milestones achieved in PET design so far, further sensitivity improvements aim to optimise factors such as the dose, throughput, and detection of small lesions. While several longer axial field-of-view (aFOV) PET systems based on pixelated detectors have been installed, continuous monolithic scintillation detectors recently gained increased attention due to their depth of interaction capability and superior intrinsic resolution. As a result, the aim of this work is to present and evaluate the performance of two long aFOV, monolithic LYSO-based PET scanner designs.

**Methods:**

Geant4 Application for Tomographic Emission (GATE) v9.1 was used to perform the simulations. Scanner designs A and B have an aFOV of 36.2 cm (7 rings) and 72.6 cm (14 rings), respectively, with 40 detector modules per ring each and a bore diameter of 70 cm. Each module is a 50 × 50 × 16 mm^3^ monolithic LYSO crystal. Sensitivity, noise equivalent count rate (NECR), scatter fraction, spatial resolution, and image quality tests were performed based on NEMA NU-2018 standards.

**Results:**

The sensitivity of design A was calculated to be 29.2 kcps/MBq at the centre and 27 kcps/MBq at 10 cm radial offset; similarly, the sensitivity of design B was found to be 106.8 kcps/MBq and 98.3 kcps/MBq at 10 cm radial offset. NECR peaks were reached at activity concentrations beyond the range of activities used for clinical studies. In terms of spatial resolution, the values for the point sources were below 2 mm for the radial, tangential, and axial full width half maximum. The contrast recovery coefficient ranged from 53% for design B and 4:1 contrast ratio to 90% for design A and 8:1 ratio, with a reasonably low background variability.

**Conclusions:**

Longer aFOV PET designs using monolithic LYSO have superior spatial resolution compared to current pixelated total-body PET (TB-PET) scanners. These systems combine high sensitivity with improved contrast recovery.

## Background

Over the last 3 decades, positron emission tomography (PET) has achieved several milestones, with the introduction of fully 3D acquisitions [1], attenuation correction from CT images [2], and, more recently, the time-of-flight (TOF) detection [3, 4] and silicon photomultiplier (SiPM)-based detector developments [5]. However, further sensitivity improvements enable low dose, higher throughput, total-body dynamic imaging, and the detection of smaller lesions.

A longer scanner geometry inherently has a higher sensitivity. Although the idea of extended axial field-of-view (aFOV) scanners dates to the early 1990s and some first prototypes were built in the early 2000s [6], due to many technical challenges, a total-body PET (TB-PET) based on the most recent detector technology was realised only recently [7]. The EXPLORER consortium, a collaboration between the University of California, Davis (UC Davis), United Imaging Healthcare Shanghai, and the University of Pennsylvania, resulted in the first TB-PET systems. The PennPET Explorer, whose aFOV has been expanded from 64 to 143 cm, uses pixelated 3.86-mm LYSO crystals and achieves a coincidence time resolution (CTR) of 245 ps and a tangential/radial spatial resolution of 4 mm at the centre [8]. The uExplorer PET/CT scanner (the first installed TB-PET at UC Davis) has an aFOV of 194 cm and 505 ps TOF. It is based on LYSO crystals of 2.76 mm width. The reported spatial resolution ranges from 3 mm at the centre of the axial and transverse FOV to 4.7 mm at a radial and axial offset [9]. Both systems are highly sensitive given their extended axial length; while the PennPET Explorer emphasises improving TOF resolution, the uExplorer achieves a better spatial resolution and higher volume sensitivity. Recently, the Biograph Vision Quadra PET/CT system was introduced as a commercially available TB-PET system with an aFOV of 106 cm based on the 26.3 cm Biograph Vision 600 PET/CT system technology. Both systems have a similar spatial resolution (ranging from 3.3 to 3.8 mm) and TOF resolution (210 ps for the Biograph Vision and 230 ps for the Quadra), but the Biograph Vision Quadra exhibits a much higher sensitivity due to the more extended axial coverage [10, 11]. Very recently, the Omni Legend digital PET/CT System of 32 cm aFOV based on BGO detectors has been introduced, with promising sensitivity and lesion detectability. Preliminary results reported a spatial resolution ranging from 3.5 to 4.5 mm using analytical and iterative reconstruction techniques [12].

While the optimal axial length of the TB-PET system depends on the application, the increase in geometric coverage by additional detectors comes with a considerably higher cost [13]. Furthermore, extending the aFOV to improve the system sensitivity and, consequently, other properties, such as the system spatial resolution, has a countereffect on the latter. Due to the more oblique gamma rays, the parallax effect is more pronounced in PET scanners with a very long axial extent when the depth-of-interaction (DOI) information in the crystal is not available [14] or challenging to extract, such as in pixelated detectors [15]. Another limitation of the pixelated crystals is their size. While a smaller crystal width leads to a better intrinsic detector resolution, other parameters such as sensitivity, timing resolution, and cost are compromised [16].

Therefore, continuous monolithic scintillation detectors are drawing more interest with their DOI capability and are an attractive alternative to conventional pixelated PET detectors [17]. In terms of sensitivity, the presence of DOI information prevents parallax errors, which allows for the axial extension of the FOV and the employment of thicker crystals. This, together with the absence of cuts in monolithic crystals, presents a significant improvement in the detector and geometric sensitivity. Moreover, it has been shown that the DOI information partially corrects for the parallax effect and improves the radial resolution [18]. Monolithic detector technology has been used recently in several commercial small animal scanners. Monolithic LYSO crystals of 25.4 mm × 25.4 mm × 8 mm have been employed first in pre-clinical scanners, and a 1 mm^3^ volumetric spatial resolution was reported for the β-cube commercially available preclinical PET scanner [19]. These detectors are optimised towards superior spatial resolution for a small bore (mice and rats). The performance of monolithic scintillation crystals of tens of millimetres in width and different thicknesses has been investigated by different groups to show the potential of using such detectors in clinical PET scanners. A monolithic 22-mm-thick LYSO detector achieved a spatial resolution of 1.7 mm full width half maximum (FWHM) with readout from the back side only [20] and 1.1 mm FWHM with dual-sided readout [21], while the measured spatial resolution of a 15-mm LYSO thick crystal is 1.8 mm [22].

However, the two main challenges of monolithic scintillators are the lengthy calibration procedure required for event positioning and the timing estimation due to spreading the scintillation light over multiple SiPMs. To address the former challenge, new calibration methods were developed to accelerate the calibration procedure while achieving a spatial resolution of 1.1 mm FWHM for 10-mm-thick LSO:Ce crystals [23] and 1.4 mm FWHM for a 12-mm LYSO crystal [24]. Further improvement in the spatial resolution of a 50 mm × 50 mm × 16 mm LYSO detector with six layers of DOI reported values of 1.14 and 1.17 mm FWHM for the whole detector with the artificial neural network and a mean nearest neighbour (MNN) positioning algorithms, respectively [25]. As for the latter challenge, simulation and experimental tests using AI-based algorithms show a CTR below 200 ps for an 8-mm LYSO monolith [26]. Higher CTR values are expected for thicker crystals due to the light spreading, although promising simulation results for 16-mm-thick LYSO crystals using convolutional neural networks (CNNs) reported a CTR of 141 ps FWHM [27].

As for clinical applications, a simulation study considered different paediatric PET designs in axial lengths and monolithic crystal thicknesses (22 mm and 11 mm) and showed that a system spatial resolution of around 2 mm could be achieved [28]. Another Monte Carlo simulation study showed the superior quality of the images produced with a whole-body clinical TOF-PET ring based on 32 mm × 32 mm × 22 mm LYSO: Ce crystals with dual-sided readout [29].

Having laid out the characteristics of the current TB-PET systems and the advantages and challenges of using monolithic crystals, this work aims to present two simulated medium to long aFOV, monolithic LYSO-based PET scanner designs, that achieve high sensitivity and superior spatial resolution while being cost-effective. The first design, referred to as design A in the remainder of this paper, has an axial extent of ~ 35 cm, employs 50 mm × 50 mm × 16 mm LYSO crystals, and presents a gain in sensitivity compared to the current state-of-the-art PET systems (15–30 cm axial length) while maintaining a reasonable cost given the moderate axial extension. Design B consists of two adjacent designs A, to further increase the axial coverage for body imaging applications.

We evaluate the performance of both simulated systems in terms of sensitivity, count rate, spatial resolution, and image quality.

## Methods

### Simulated scanner designs

**Fig. 1:**
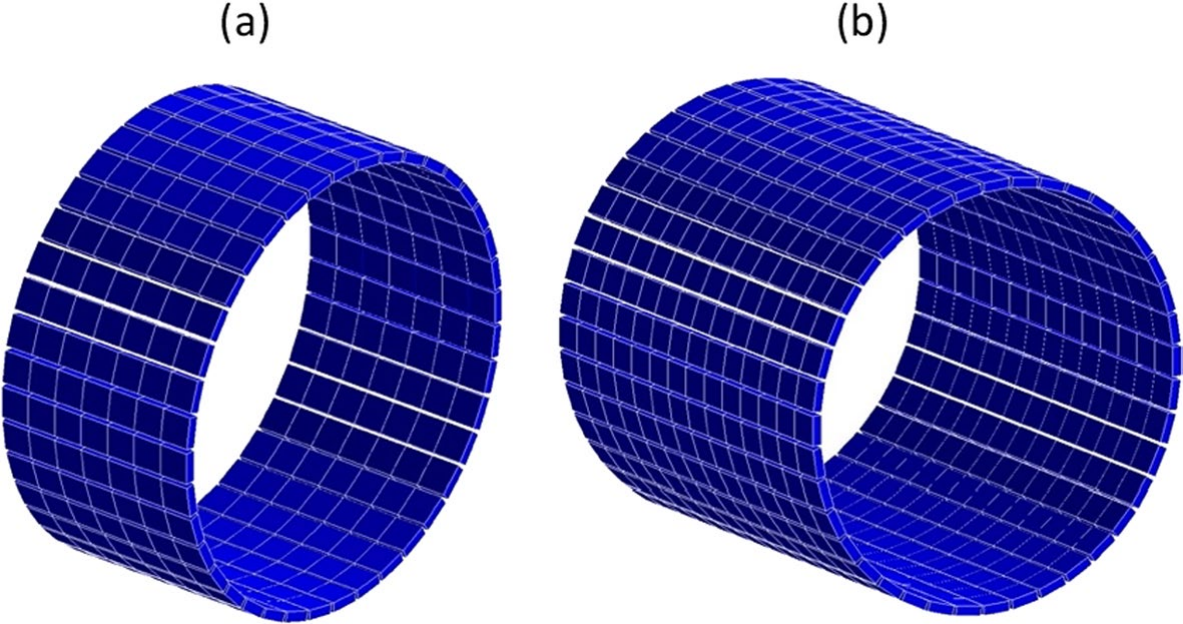
GATE visualised **a** design A of 36.2 cm aFOV; **b** design B of 72.6 cm aFOV

All simulations were conducted using the Geant4 Application for Tomographic Emission (GATE) v9.1. The A and B scanner designs under study have an aFOV of 36.2 cm and 72.6 cm, with opening angles of 27° and 46°, respectively. Both designs consist of 40 detector modules per ring, resulting in a bore diameter of 70 cm. This is a smaller bore than conventional pixelated PET scanners. The scanner diameter is chosen smaller since monolithic detectors have intrinsic DOI. Each module is composed of a monolithic 50 × 50 × 16 mm^3^ LYSO crystal. Design A has 7 of these rings (Fig. 1a) with a gap of 2 mm between every two consecutive rings bringing the total scanner length to 36.2 cm, while design B has 14 rings (Fig. 1b). The scanners were modelled with a 200 ps CTR, 3 ns coincidence window, and an 11.5% energy resolution (440–650 keV window). The dead time model was set to paralysable 300 ns based on experimental measurements of monolithic detectors from the β-cube of MOLECUBES [19]. The simulations were performed using the NEMA NU-2018 standards [30] for sensitivity, count rate performance, spatial resolution, and image quality measurements.

#### Sensitivity

Sensitivity was tested with a 5 MBq line source (70 cm long) of 511 keV back-to-back gammas with no surrounding material placed at the centre of the scanner and 10 cm radial offset from the centre. The scan time was 30 s, and the root output was processed to sort out true, scatter, and random events. The sensitivity was calculated as

1 $${\text{Sensitivity}} = \frac{{{\text{True }}\;{\text{counts}}\;{\text{ detected}}}}{{{\text{Activity}}\times \;{\text{Acquision time}}}}$$

#### Count rate performance

The count rate performance is simulated using an ^18^F positron source (70 cm long) placed at a radial offset of 4.5 cm from the axis of the tomograph. The activity of the line source was changed from 0.045 to 50 kBq/mL while ensuring that each simulation has a minimum of one million prompt counts. Sinogram-based NEMA specifications were used to extract the true, scatter, random, and noise equivalent count rate (NECR) from the prompts datasets [31]. NECR was calculated using Eq. 2 [32]:

2 $${\text{NECR}} = \frac{{T^{2} }}{S + T + R}$$

*T*, *S*, and *R* are true, scatter, and random coincidence count rates, respectively.

The scatter fraction (SF), defined by NEMA as the ratio of scatter to scatter and true counts, was calculated at low activity using Eq. 3:

3 $${\text{SF}} = \frac{S}{S + T}$$

#### Spatial resolution

An ^18^F positron point source of 3.7 MBq total activity was used to evaluate the system’s spatial resolution. It is placed into a 0.5 mm water sphere enclosed into a cylindrical glass capillary with 0.52 inner diameter, 1.8 mm outer diameter, and 0.9 mm height. The source is imaged at multiple radial positions (1, 10, and 20 cm) from the centre of the FOV and two axial positions (the centre of the aFOV and at three-eighths from the centre of the aFOV). At least one million coincidences were collected per source position.

The simulation output is post-processed to include the intrinsic detector resolution by blurring the line of responses (LOR) endpoints based on 50 × 50 × 16 mm^3^ LYSO performance measurements [25]. Simulated data were reconstructed using the quantitative emission tomography iterative reconstruction (QETIR) software developed at Ghent University and used in simulation studies to reconstruct data from the total-body J-PET scanner [33–35]. Maximum Likelihood Estimation Method (MLEM) algorithm with ten iterations and no subsets was used. The sensitivity map was also generated in QETIR, and the voxel size used is 0.5 mm in each direction. For each source position, axial, radial, and tangential resolutions are estimated by determining the FWHM of the point spread function (PSF) in all three directions.

#### Image quality

The NEMA image quality (IQ) phantom with six hot spheres of different diameters (10, 13, 17, 22, 28, and 37 mm) inserted in a body phantom with non-uniform attenuation was simulated for 400 s. Two sphere-to-background ratios (SBR) were used, 4:1 and 8:1, with a background activity concentration of 5.3 kBq/mL. The true counts were reconstructed into 2-mm voxel images using the TOF-MLEM algorithm in QETIR (twenty iterations and no subsets) with a TOF resolution of 200 ps. Attenuation correction was applied by generating a density map with a C++ script. The contrast recovery coefficients (CRCs) were calculated as per the NEMA specifications, defined below:

4 $${\text{CRC}} = \frac{{C_{H} {/}C_{B} - 1}}{{A_{H} {/}A_{B} - 1}}$$

where $C_{{\text{H}}}$ is the average counts in the region of interest (ROI) drawn around each of the hot spheres and $C_{{\text{B}}}$ is the average of the background ROI counts, while $A_{{\text{H}}}$ is the activity concentration of the hot spheres, and $A_{{\text{B}}}$ is that of the background spheres. According to the NEMA specifications, sixty background ROIs of each size were drawn, twelve on each of the following slices: the central slice and two slices on either side at + 2 cm, + 1 cm, − 1 cm, and − 2 cm. The background variability *N*_*j*_ for each sphere j of the six different diameters was also calculated as:

5 $$N_{j} = \frac{{{\text{SD}}_{j} }}{{C_{B} }}$$

SD_*j*_ is the standard deviation of the background ROI counts for sphere *j* considering all sixty background ROIs.

## Results

### Sensitivity

**Fig. 2:**
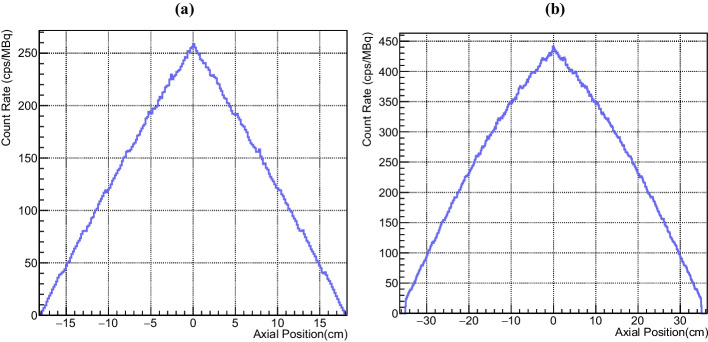
Axial sensitivity profiles for the 0 cm radial offset position for **a** design A and **b** design B with a bin width of 1.65 mm

The calculated sensitivity for design A is 29.2 kcps/MBq at the centre and 27 kcps/MBq at 10 cm radial offset. Design B achieves a sensitivity of 106.8 kcps/MBq at the centre and 98.3 kcps/MBq at 10 cm off-centre. The axial sensitivity profiles of both designs at 0 cm radial offset are shown in Fig. 2. Table 1 describes and compares the simulated NEMA sensitivity values and geometric characteristics of designs A and B, the Biograph Vision 600 and Quadra.

**Table 1:** Simulated NEMA sensitivity and geometric characteristics comparison between designs A and B and the biograph vision 600 and biograph Quadra

	Design A	Design B	Biograph vision 600	Biograph vision Quadra
NEMA sensitivity (kcps/MBq)	28.1	102.6	20.2	232.5 (MRD 322)–110.4 (MRD 85)
Axial length (cm)	36.2	72.6	26.1	106
Crystal thickness (mm)	16 (LYSO)	16 (LYSO)	20 (LSO)	20 (LSO)
Detector surface (× 10^6^ mm^2^)	0.70	1.40	0.62	2.49
Scintillator volume (× 10^6^ mm^3^)	11.20	22.40	12.45	49.80

### Count rate performance

**Fig. 3:**
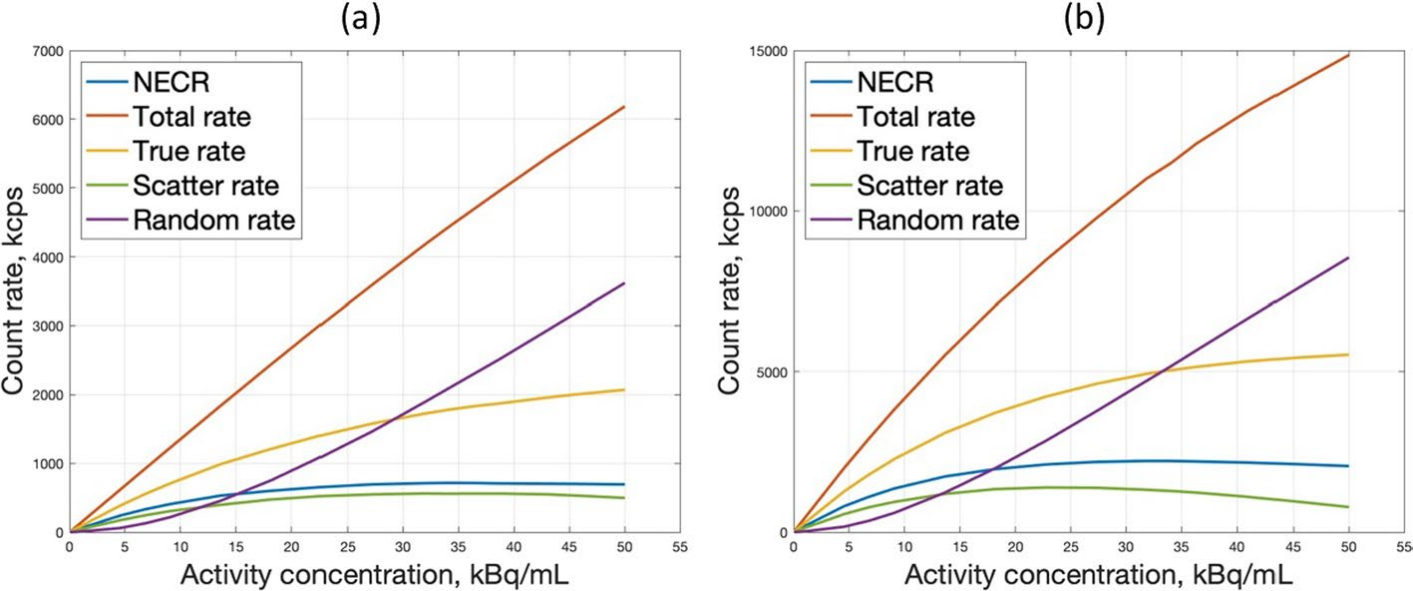
Count-rate performance with 70-cm line source for **a** design A and **b** design B

Figure 3 shows the count rate plots for true, random, scatter counts, and NECR for both designs. The peaks NECR are 716 kcps for design A and 1235 kcps for design B at 34 kBq/mL both. At higher activities, the random rate increases while the value of the NECR decreases.

The calculated scatter fraction is 32% for both designs. It is stable over a range of activity at which the random rates are negligible (below 1% of the trues rate based on NEMA).

### Spatial resolution

Tables 2 and 3 show the spatial resolution results for both designs. These results include the detector spatial blurring effects and the contribution of the positron range and acolinearity that were simulated in GATE. The radial, tangential, and axial FWHM values for the point sources at different radial and axial positions are below 2 mm with minimal variations across the transverse and axial FOV. It is worth mentioning that iterative reconstruction methods show superior results to analytical algorithms such as filtered back projection (FBP). The iterative reconstruction method does, however, not include any modelling.

**Table 2:** Spatial resolution FWHM for design A

	Radius (cm)	Radial (mm)	Tangential (mm)	Axial (mm)
aFOV centre	1	1.44	1.36	1.45
	10	1.46	1.41	1.25
	20	1.48	1.38	1.23
3/8 aFOV	1	1.34	1.32	1.23
	10	1.41	1.38	1.24
	20	1.41	1.26	1.14

**Table 3:** Spatial resolution FWHM for design B

	Radius (cm)	Radial (mm)	Tangential (mm)	Axial (mm)
aFOV centre	1	1.45	1.48	1.36
	10	1.46	1.44	1.32
	20	1.5	1.34	1.24
3/8 aFOV	1	1.37	1.37	1.31
	10	1.39	1.35	1.20
	20	1.44	1.31	1.17

### Image quality

**Fig. 4:**
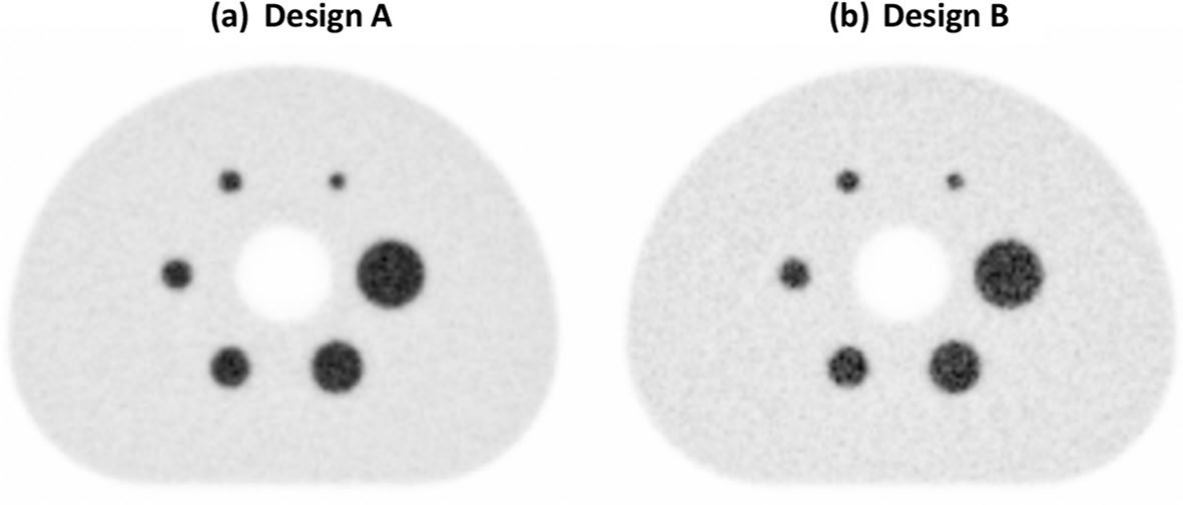
Central slice of the 10th iteration of the reconstructed image of **a** design A and **b** design B, the sphere-to-background ratio is 8:1 and the data acquisition time is 400 s

The reconstructed NEMA IQ phantom for designs A and B for a sphere-to-background concentration ratio of 8:1 is shown in Fig. 4.

**Fig. 5:**
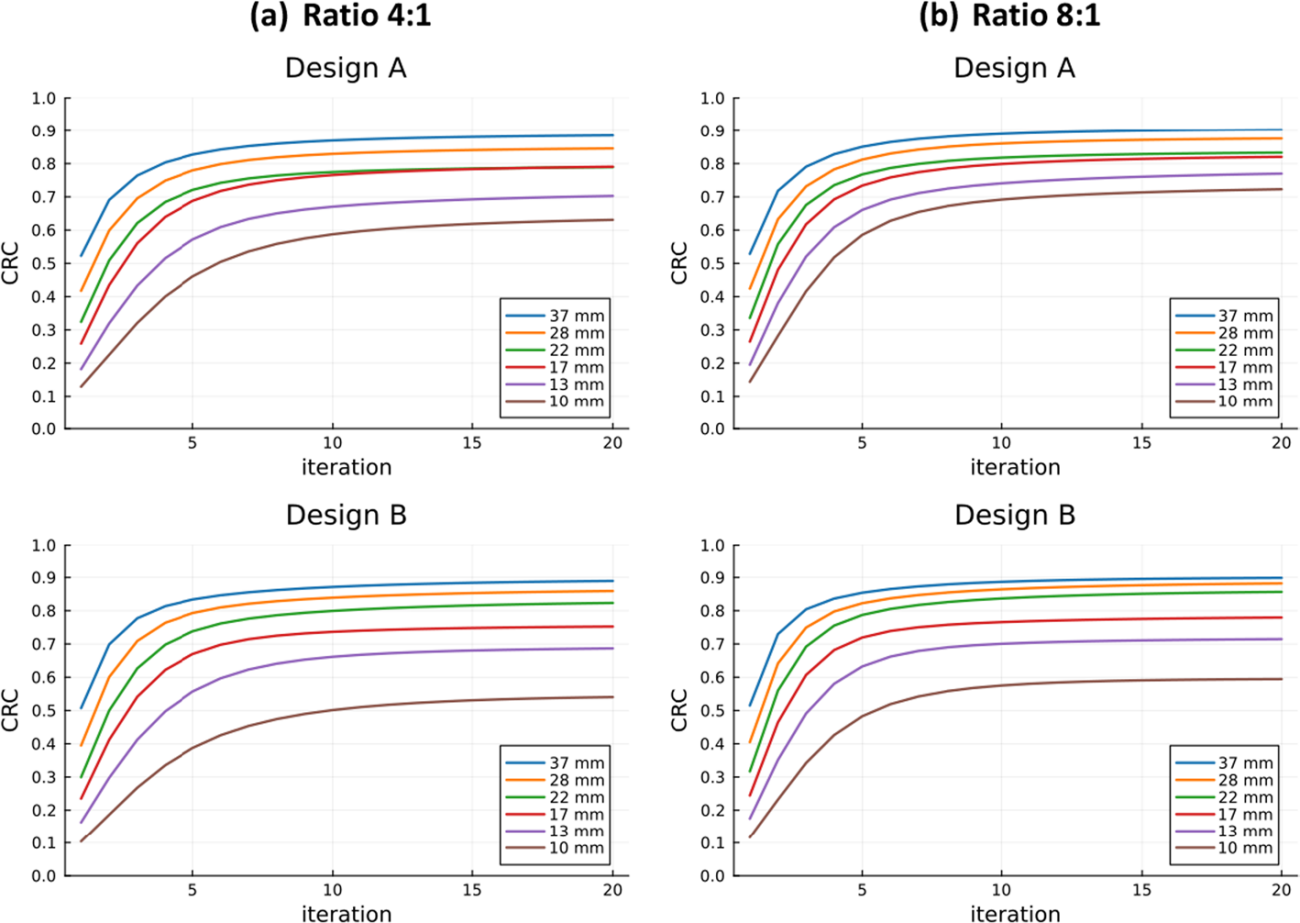
Contrast recovery coefficient (CRC) values of the IQ phantom for both designs with **a** 4:1 contrast and **b** 8:1 contrast

Figure 5 shows the CRC curves for both designs and contrast ratios as a function of the iteration number. For all four plots, the CRC values improve with higher iteration numbers up until iteration 10, after which the changes are minimal. For design A, the CRC values for the 4:1 ratio range from 60 to 90% as the sphere size increases. The 8:1 ratio shows improved CRCs, especially for the smaller spheres. The same trend between both ratios was observed for design B but with slightly lower CRC values than design A for the smaller spheres.

**Fig. 6:**
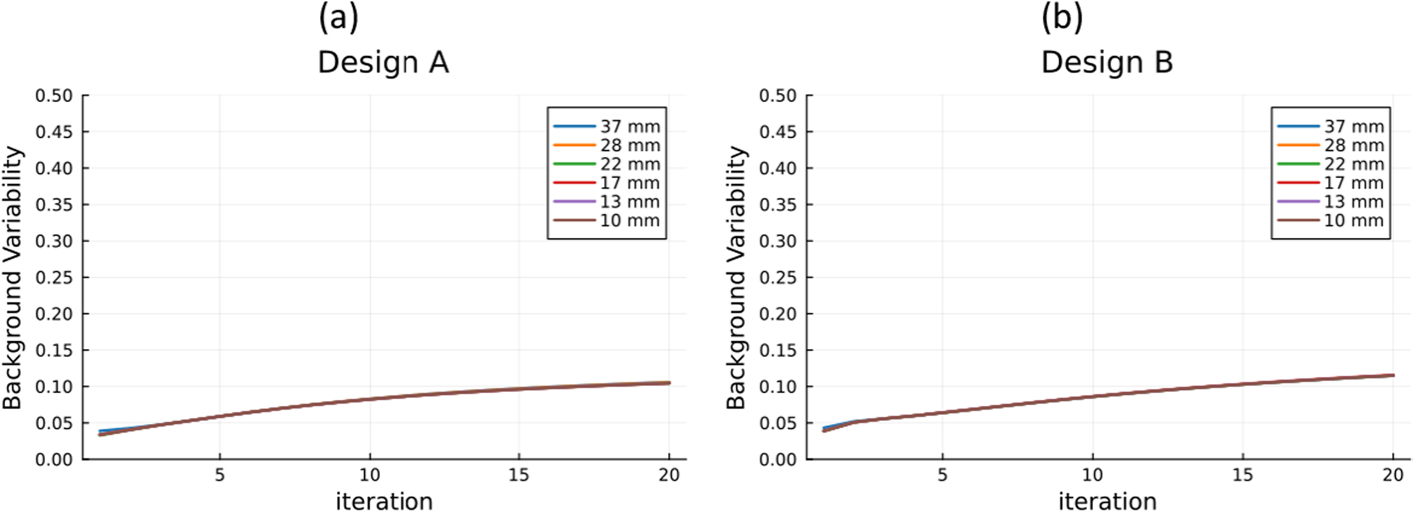
Background variability curves for sphere-to-background concentration ratios of 4:1 for **a** design A and **b** design B

The plots of the background variability as a function of the iteration number for designs A and B with a 4:1 contrast ratio (Fig. 6) show a higher variability with higher iteration numbers independently of the sphere size. The same trend was observed with similar values for the 8:1 contrast ratio.

## Discussion

Design A, with an aFOV of 36.2 cm, achieves a sensitivity that is 39% higher than the simulated sensitivity of the Biograph Vision 600 as shown in Table 1. Compared to the conventional PET/CT, this moderate extension in the aFOV provides a noticeable sensitivity improvement that is favourable for certain applications, such as organ imaging, while it does not result in a significantly higher cost as in other TB-PET systems. The results also show a nearly fourfold increase in sensitivity between designs A and B when doubling the aFOV, which is explained by the increase in the geometric efficiency and the coincidence detection efficiency [36].

Design B is a more attractive option for TB-PET applications requiring longer aFOV to maximise the sensitivity for different clinical and research applications [13]. It achieves a comparable average system sensitivity to the Biograph Quadra in MRD 85 mode.

It is important to mention that the sensitivity gain in a long aFOV PET scanner depends on the axial extent of the source. It has been shown that for a single point source that mimics single-organ imaging, the primary gain in sensitivity is in the first 50 cm–1 m. Also, in realistic imaging situations where the imaged object has a medium to large diameter, the attenuation of 511 keV in the object/body is more pronounced for longer aFOV. LORs at larger oblique angles will result in a reduced fraction of detected photons, negatively affecting the sensitivity [7]. Therefore, a scanner with a medium aFOV (around 70 cm, like design B) covers the torso and ensures a high system sensitivity for both single-organ and partial torso imaging while being cost-effective. In most clinical PET-CT studies, imaging is only required from the thighs to the start of the brain.

It is also shown in Table 1 that design A has slightly less scintillator volume and 13% more detector surface than the Biograph Vision 600 since it has a longer aFOV but also a smaller bore diameter. While more detector surface is needed for the Biograph Vision 600 in the transaxial FOV to cover the perimeter of the 78 cm bore diameter, the larger volume of scintillators is due to the thicker crystals used. The trade-off between scanner aFOV and crystal thickness was investigated in previous work [37], and it was concluded that using shorter crystals in longer aFOV scanners can be beneficial. Moreover, design B, which aFOV is 68% that of the Quadra, employs less than half of the scintillator volume of the Quadra and achieves a sensitivity value comparable to the Quadra in MRD 85.

The count rate performance is an essential NEMA measure to account for the noise effects of subtracting the random and scatter counts and the ability of the PET scanner to measure highly radioactive sources [32]. It is important to mention that the NECR curve depends on the dead-time model used in the simulation. In Fig. 3, the true count rate is linear up to an activity of 5 kBq/mL, which covers the range of activity for a clinical ^18^F-FDG study. Also, the NECR peaks are reached at 34 kBq/mL activity concentration value which is above the concentrations considered in the clinical studies [38].

The spatial resolution is the most important performance attributed to both designs, given the choice of monolithic LYSO crystals with multi-layer DOI. The physics of positron decay and annihilation imposes a limit on the achievable spatial resolution for PET scanners [39]. This highlights the importance of choosing a detector that can achieve a high intrinsic spatial resolution and minimally contributes to the system's spatial resolution degradation. The FWHM values show a flat sub-2 mm spatial resolution across the axial/transverse FOV, allowing the detection of small lesions and abnormalities. Compared to the human PET scanner with the highest resolution (uExplorer, 3 mm in the centre and 4.7 mm at 20 cm), we obtained around 50% improvement at the centre of the axial/transverse FOV while at off-centre points, a 2–3 times improvement was observed. This will make quantification accuracy independent of the position in the patient.

The CRC results are comparable to those reported by the digital Biograph Vision 600 and the Quadra [10, 11] and other long aFOV systems such as the PennPET Explorer and uExplorer [9, 40]. A slightly superior performance in contrast recovery was observed for design A, which could be explained by the presence of more oblique LORs in design B.

Both designs show a higher background variability when compared to the Biograph Vision 600 and the Quadra, which can be associated with the smaller acquisition time used in this work. In the PennPET Explorer performance paper [40], they study the dependence of the background variability on the acquisition time and show a significant improvement at higher acquisition times. The reconstruction parameters chosen in this work might have affected the image quality results, and a future study to optimise these parameters can be conducted.

## Conclusions

In this work, designs A and B of aFOVs 36.2 cm and 72.6 cm, respectively, based on monolithic LYSO scintillators, showed superior spatial resolution compared to current long aFOV systems. Design A, which aFOV is moderately more extended than the Biograph Vision 600 but with no increase in scintillator volume, proved to be more sensitive. At the same time, the sensitivity of design B is comparable to that of the Quadra in MRD 85 at less than half of the scintillator volume. Both designs have a count rate capability that covers an activity range that extends beyond that of clinical regimes and a stable scatter fraction of 32%. Image quality results are promising, with CRC values reaching 90% and acceptable background variability.

## Data Availability

The datasets used and/or analysed during the current study are available from the corresponding author upon reasonable request.

## Abbreviations

PET: Positron emission tomography
CT: Computed tomography
TOF: Time of flight
SiPM: Silicon photomultiplier
aFOV: Axial field of view
TB-PET: Total-body PET
CTR: Coincidence time resolution
NEMA: National Electrical Manufacturers Association
DOI: Depth of interaction
FWHM: Full width at half maximum
GATE: Geant4 Application for Tomographic Emission
NECR: Noise equivalent count rate
SF: Scatter fraction
MRD: Maximum ring difference
LOR: Line of response
QETIR: Quantitative emission tomography iterative reconstruction
MLEM: Maximum-likelihood expectation–maximisation
PSF: Point spread function
IQ: Image quality
SBR: Sphere-to-background ratio
CRC: Contrast recovery coefficient
ROI: Region of interest
SD: Standard deviation
FBP: Filtered back projection
